# Neurophysiology of Gaze Direction as Poly-Equilibrium

**DOI:** 10.3390/neurosci6030085

**Published:** 2025-09-04

**Authors:** Laurent Goffart

**Affiliations:** Centre Gilles Gaston Granger, UMR 7304 Centre National de la Recherche Scientifique, Aix Marseille Université, 13621 Aix-en-Provence, France; laurent.goffart@cnrs.fr

**Keywords:** fixation, saccade, pursuit, neurophysiology, neuro-ophthalmology, brainstem, cerebellum, extraocular muscles, equilibrium, symmetry breaking

## Abstract

The static orientation of the eyes during visual fixation is determined by the simultaneous operation of multiple equilibria. This phenomenon is collectively referred to as poly-equilibrium, which involves multiple systems that work together to cancel each other out and establish gaze direction. While other systems, such as audio- and cervico-ocular systems, may also contribute to gaze direction, this review focuses primarily on the commands issued by the vestibulo- and visuo-oculomotor systems that determine gaze direction, as they play a key role in the poly-equilibrium process. From the visual and vestibular activities accompanying the appearance of an object in the central visual field to the recruitment of premotor neurons responsible for the generation of slow and saccadic eye movements, a delicate balance is maintained. As long as the recruited channels convey commands that counterbalance each other, no movement is initiated. This alternative viewpoint leads to reconsidering the nature of saccadic and pursuit eye movements. Rather than viewing them as the dynamic reduction in brain signals encoding kinematic parameters such as position or velocity, they can be seen as the physical expression of intracerebral processes restoring balanced activities between sensorimotor channels whose recruitment leads to mutually opposed movements.

## 1. Introduction

Contrary to the statement that “when the eyes are in their primary position, the lateral and medial recti are mostly relaxed and the mechanical forces of the socket can keep the eye stable in this position”, the orientation of each eyeball is actually determined by the tensions that six extraocular muscles exert upon it. For each muscle, the tension is determined by the continuous influx of action potentials emitted by the group of motor neurons that innervate its fibers. The eyes change their orientation as soon as modifications in the firing rate of motoneurons cause unbalanced changes in the tension of extraocular muscles. The movement stops when the tensions mutually cancel each other out, when a new equilibrium state is restored. 

This continuous and antagonistic drive of the contraction of extraocular muscles was first highlighted by the demonstration that an eye movement could still result from the additional relaxation of an extraocular muscle, even after the agonist muscle’s action was neutralized [1]. We shall see in the next section that subsequent recordings of motoneurons’ activity confirmed their sustained and relatively high firing rate when the eyes are centered in the orbits and directed straight ahead.

**Figure 1:**
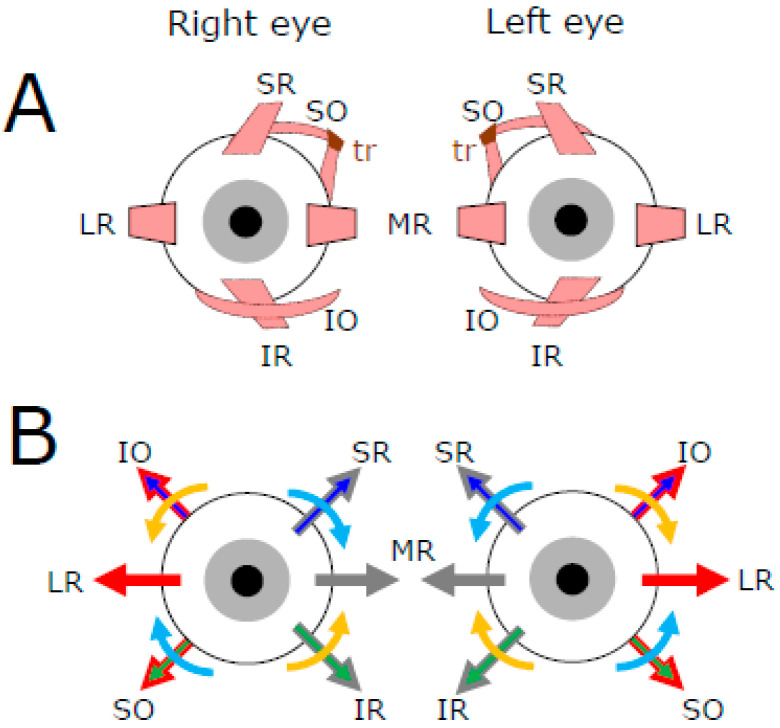
Schematic representation of the extraocular muscles (**A**) and pulling directions engaged by the contraction of their fibers when the eyes are centered in the orbit (**B**). LR: lateral rectus, MR: medial rectus, SR: superior rectus, IR: inferior rectus, SO: superior oblique, IO: inferior oblique, tr: trochlea. The red arrows indicate the muscles whose contraction abducts the eye, and the grey arrows indicate the muscles whose contraction adducts the eye. The blue arrows indicate the muscles whose contraction elevates the eye (supraduction), and the green arrows indicate those whose contraction directs the eye downward (infraduction). The turquoise arrows indicate the muscles whose contraction causes an incycloduction of the eye, and the orange arrows indicate those whose contraction causes an excycloduction. Figure modified and upgraded from [3].

The approximate location of extra-ocular muscles is illustrated in panel A of Figure 1. Four rectus muscles attach to the sclera anteriorly with respect to the eyeball equator. When they contract, they pull the eye toward the side where the muscle is attached, contrary to the contraction of the oblique muscles whose insertions are posterior to the equator. The primary actions of the lateral (LR) and medial (MR) rectus muscles are antagonistic to each other because their insertions are located on opposite sides of each eyeball. The contraction of LR fibers rotates the eyeball toward the temporal side (a movement called abduction) if it is associated with the relaxation of MR fibers. Conversely, the contraction of MR fibers rotates the eyeball toward the nasal side (adduction) if it is associated with the relaxation of LR fibers. In the absence of relaxation of the antagonist muscle, the co-contraction of both lateral and medial rectus muscles would retract the eyeball within the orbit. Thus, with the head upright and gaze directed straight ahead, a movement of both eyes toward the right results from the contraction of the right LR and left MR combined with the relaxation of the right MR and left LR. Provided that the tensions exerted by the four other muscles do not change, the direction of gaze will change and move along the plane passing through the LR and MR muscles.

Still with the gaze directed straight ahead, the contraction of the superior rectus (SR) muscles causes supraduction (also called elevation), incycloduction (intorsion), and adduction of the eyeball, whereas the contraction of the inferior rectus (IR) muscles causes its infraduction (depression), excycloduction (extorsion), and adduction (Figure 1B). The contraction of inferior oblique (IO) muscles causes excycloduction, supraduction, and abduction, whereas the contraction of superior oblique (SO) muscles causes incycloduction, infraduction, and abduction of the eye [2]. Thus, the combined contraction of SR and IO directs the gaze upward while the incycloduction and adduction caused by SR contraction balance the excycloduction and abduction caused by the IO contraction. Likewise, the combined contraction of IR and SO directs the gaze downward while the excycloduction and adduction caused by IR contraction balance the incycloduction and abduction caused by the SO contraction.

By convention, the eye movements are described as rotations about three virtual axes: a vertical axis for horizontal (leftward and rightward) movements, a horizontal axis for vertical (upward and downward) movements, and an anteroposterior axis for torsional movements (intorsion and extorsion). These rotations rely on synergies between anatomo-physiological elements that are invisible to investigations limited to kinematic or dynamic studies. However, these macroscopic analyses reveal some constraints to the contractions of extraocular muscles. For example, kinematic studies of healthy subjects with their head held upright have shown that a single axis of rotation is sufficient to describe the eye movements during slow pursuit movements and very rapid responses (called saccades) to visual targets. When the eye is directed straight ahead, the axis of rotation lies in a plane, called Listing’s plane, which is perpendicular to the direction of gaze [4]. Thus, visually guided rotations of the eyes seem to be constrained about the vertical and horizontal axes. However, this restriction of rotation axes to Listing’s plane is neither inviolable nor perfectly satisfied [5]. Torsions can also be observed in patients suffering from specific brain damage [6,7,8] or when a healthy subject moves the eyes with the head tilted [9]. Finally, with training, human subjects can learn to voluntarily perform torsional movements of the eyeballs [10].

## 2. Motoneuronal Control of Extraocular Muscles Contraction

**Figure 2:**
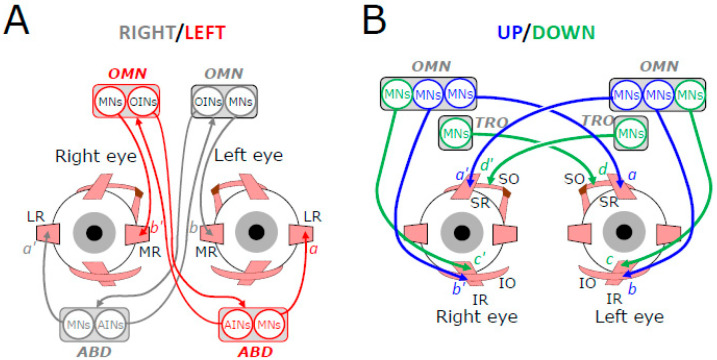
Schematic representation of the motoneuronal control of extraocular muscles’ contraction for horizontal (**A**) and vertical (**B**) eye movements. Connecting lines ended by an arrow indicate excitatory connections; those ended by a circle indicate inhibitory synaptic connections. From top to bottom, OMN: oculomotor nucleus, MNs: motoneurons, OINs: oculomotor internuclear neurons; LR: lateral rectus, MR: medial rectus, SR: superior rectus, SO: superior oblique, IR: inferior rectus, IO: inferior oblique, AINs: abducens internuclear neurons.

The motor neurons innervating the extraocular muscles are grouped in the brainstem, within three pairs of nuclei that are situated bilaterally with respect to the mid-sagittal plane [11]. The motoneurons innervating the LR muscle fibers are located in the abducens nuclei, those innervating the SO muscle fibers in the trochlear nucleus, and those innervating the fibers of MR, SR, IR, and IO in distinct sectors of the oculomotor nuclei (Figure 2).

The motoneurons supply the muscle fibers with a continuous influx of action potentials responsible for their tension. Each eye maintains a fixed orientation because the influence of abducens motoneuron activity on the LR fibers (synapses a and a’ in Figure 2A) counterbalances the influence that motor neurons in the oculomotor nucleus exert on the MR fibers (synapses b and b’) and vice versa. Likewise, the influence that motoneurons in both (left and right) oculomotor nuclei exert on SR (supraducting and incycloducting actions) and IO (supraducting and excycloducing actions) (left eye: synapses a and b; right eye: synapses a’ and b’) counterbalances the influence that motoneurons in the oculomotor nucleus and contralateral trochlear nucleus exert on IR (infraducting and excycloducting actions) and SO (infraducting and incycloducing actions), respectively (left eye: synapses c and d; right eye: synapses c’ and d’). Assuming that the number of muscle fibers, their contractile properties, the number of neurons and their firing rates are not identical between the antagonistic groups of motor units implies that these counterbalances depend upon a premotor control of motoneuron activity, which must also be bilateral.

Indeed, Figure 2B shows that, for each eye, an upward (or downward) deviation of the eyes involves bilateral motor units, with motoneurons located in the left and right oculomotor nuclei for looking upward, motoneurons in the ipsilateral oculomotor nucleus (OMN), and contralateral trochlear nucleus (TRO) for looking downward. By contrast, the motor units are unilateral for horizontal deviations, with motor neurons innervating the LR located in the abducens nucleus (ABD) and those innervating the MR in the ipsilateral OMN (Figure 1A). The motor control of horizontal eye movements becomes bilateral and binocular because of the involvement of internuclear neurons in the ABD and projecting to the contralateral OMN (AINs), but also in the OMN, projecting to the contralateral ABD (OINs) [12]. 

The last important point to keep in mind is that the motoneurons exhibit a sustained activity when the eyes are in the primary position. For some of them, the firing rate can exceed 150 spikes per second (for a summary of average and range values, see [13]; see also [14,15] for subsequent additions). This sustained activity is due to intrinsic properties of motor neurons, but also to afferent inputs from the vestibular, visual, and neck proprioceptive systems. 

We shall not review all of them but focus our attention on the inputs from vestibular neurons, which are excitatory from the contralateral vestibular nuclei and inhibitory from other ipsilateral and contralateral neurons. Then, we shall describe how visual signals contribute to the direction of gaze.

## 3. Vestibular Inputs to Extraocular Motoneurons

Among multiple other functions [16,17,18], the system of vestibular neurons is primarily involved in deviating the eyes in the direction opposite to the head rotation. This reaction, called vestibulo-ocular response (VOR), starts with sensory cells in three semicircular canals (lateral, anterior, and posterior) buried within the temporal bone. Each semicircular canal is a semi-torus-shaped structure filled with endolymphatic fluid that mechanically deflects the stereocilia of hair cells in response to accelerating or decelerating head rotations. The deflection of stereocilia either increases or decreases the discharge of primary vestibular neurons with a rate proportional to head velocity. Then, secondary vestibular neurons are located in the medial vestibular nucleus (MVN) and superior vestibular nucleus (SVN). Excitatory MVN neurons mediate vestibular signals from all canals to extraocular motoneurons involved in counter-rotating the eyes in the orbits. Inhibitory neurons in the MVN mediate vestibular signals from lateral canals to ipsilateral extraocular motoneurons, preventing them from firing. In the SVN, inhibitory neurons mediate the suppression of extraocular motoneurons involved in vertical VORs during the stimulation of anterior or posterior canals [19]. Finally, all parts of the MVN and areas of the SVN are interconnected bilaterally by commissural inhibitory fibers [20], such that a push–pull pattern of activity is engendered between the assemblies of neurons that are connected with reciprocal pairs of semicircular canals.

### 3.1. Inputs from Lateral Semicircular Canals

Located in the left and right inner ears, the two lateral canals lie in a “plane”, which is tilted relative to Reid’s stereotactic horizontal plane, but also relative to the “plane” passing through the MR and LR muscles [21,22,23]. These canals are conventionally called “horizontal” because they are engaged primarily during head rotations about the Earth’s gravity axis. During yaw rotations of the head, the firing rate of primary vestibular neurons (PVNs) on the side toward which the nose rotates (ipsilateral side) increases while the firing rate of PVNs on the contralateral side decreases. These bilateral changes in firing rate modify the activity of post-synaptic neurons in the medial and superior vestibular nuclei [24]. On either side of the brain’s midsagittal plane, these secondary vestibular neurons fall into two distinct categories. The firing rate of type 1 neurons increases during ipsiversive rotations of the head and decreases during contraversive rotations. Some of these neurons pause during saccades and fire with a rate increasing with contralateral eye position, whereas the activity of others increases with the velocity of contraversive pursuit eye movements [24,25,26,27,28]. In the monkey, these neurons have been called “Position Vestibular Pause” (PVP) and “Eye Head Velocity” (EHV). Type 2 neurons exhibit a pattern of activity opposite to type 1 neurons, increasing during contraversive head rotations and decreasing during ipsiversive ones.

**Figure 3:**
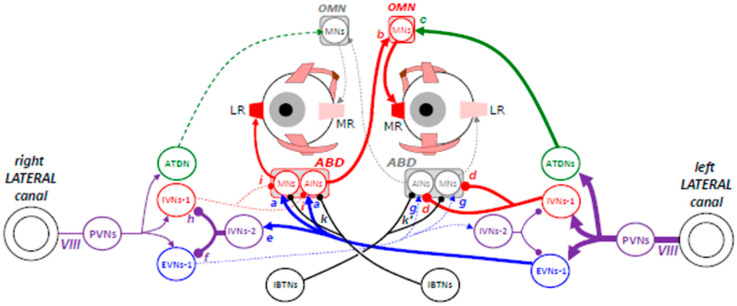
Schematic representation of the neuronal network involved in maintaining the same gaze direction while the head rotates toward the left. Connecting lines ended by an arrow indicate excitatory connections; those ended by a circle indicate inhibitory synaptic connections. The thickness of connecting lines schematizes the strength with which the neurons fire. LR: lateral rectus, MR: medial rectus, ABD: abducens nucleus, OMN: oculomotor nucleus, MNs: motor neurons, INs: internuclear neurons, PVNs: primary vestibular neurons, IVNs: inhibitory vestibular neurons, EVNs: excitatory vestibular neurons, VIII: eighth cranial nerve, IBTNs: inhibitory burst tonic and inhibitory tonic neurons. The OINs are not shown for clarity. Explanations in the main text. Figure modified and upgraded from [3].

Figure 3 schematizes the cascade of excitatory and inhibitory events leading to turning both eyes in the direction opposite to the head rotation. As the head rotates toward the left, the PVNs excite the type 1 excitatory vestibular neurons (EVNs-1) in the ipsilateral medial and ventrolateral vestibular nuclei. In turn, these EVNs-1 excite the motor (MNs) and internuclear (AINs) neurons in the contralateral abducens nucleus (blue-colored synapses a), leading to the contraction of the right LR muscle fibers. Regarding the contraction of the left MR muscle fibers, the agonist motoneurons in the OMN receive excitatory input from AINs in the contralateral abducens nucleus (red-colored synapse b) and from neurons in the ipsilateral MVN, whose axons constitute the ascending tract of Deiters (ATDNs; green-colored synapse c). 

In parallel to this chain of excitatory signals, the spikes emitted by type 1 inhibitory vestibular neurons (IVNs-1) suppress the activity of MNs and AINs innervating the antagonist muscles (red-colored synapses d). This inhibition of neurons in the ipsilateral abducens nucleus is complemented by the excitation that EVNs-1 exerts on type 2 neurons (IVNs-2) in the contralateral vestibular nuclei (blue-colored synapse e). By inhibiting the activity of EVNs-1 in the contralateral side (purple-colored synapse f), these IVNs-2 reduce the excitatory influence that contralateral EVNs-1 exert upon the motor and internuclear neurons that innervate the antagonist muscles (blue-colored synapses g). IVNs-2 also inhibit the activity of IVNs-1 (purple-colored synapse h), thus disinhibiting the cells (synapses i), which, under the influence of EVNs-1 (blue-colored synapses a), drive the counter-rotation of the eyes in their orbit.

When the eyes are static and directed straight ahead, the excitatory inputs from EVNs-1 in the contralateral vestibular nuclei (synapses a in the right abducens nucleus and synapses g in the left abducens nucleus) combine with inhibitory inputs from IVNs-1 in the ipsilateral vestibular nuclei (synapses i in the right abducens nucleus and synapses d in the left abducens nucleus). However, these excitatory and inhibitory inputs do not cancel each other out. Indeed, it has been shown that abducens neurons respond asymmetrically to an electrical microstimulation of vestibular nerves; contralateral stimulations dominate. Moreover, during VOR, the amplitude of the discharge increase evoked by contraversive rotation is consistently larger than the amplitude of the decrease during ipsiversive rotation [29]. Finally, we shall see next that the neurons in the abducens nuclei receive additional inhibitory inputs (synapses k and k’) from burst tonic and tonic neurons (IBTNs) in the contralateral medial vestibular nucleus (MVN) and nucleus prepositus hypoglossi (NPH).

Like MNs, premotor neurons in MVN exhibit a substantial sustained firing rate when the gaze is directed straight ahead and the head does not move. Thus, additional excitatory inputs to motor and internuclear neurons in the abducens nucleus (synapses a) that are not associated with additional inhibitory inputs (synapses i and k) generate a slow binocular eye movement. Likewise, less inhibitory inputs (from IVNs-1 and IBTNs) that are not associated with less excitatory input generate an ipsiversive slow binocular eye movement. In other words, slow binocular eye movements can be generated from an ipsilateral inhibition, from a contralateral vestibular excitation, or from a combination of both. Thus, the ipsilateral slow movements elicited by electrical microstimulation of Purkinje cells in the flocculus and paraflocculus may involve IVNs-1 (synapses i or d), whereas the contralateral slow movements evoked by microstimulation in the vestibular nuclei [30] likely involve the recruitment of axons of EVNs-1 (synapses a or g). When it is applied in the nearby nucleus prepositus hypoglossi (NPH), an electrical microstimulation evokes an ipsilateral slow eye movement [30,31], instead of a contralateral one. Cannon and Robinson explained that the movement results from recruiting the axons of contralateral EVNs-1 and ipsilateral IVNs-2. Recruiting the former would excite motor and internuclear neurons in the ipsilateral abducens nucleus, whereas recruiting the latter would disinhibit them (by inhibiting the ipsilateral IVN-1) while inhibiting the ipsilateral EVNs-1 [31]. However, it may also be that the microstimulation recruits burst tonic neurons projecting to the contralateral abducens nucleus. Indeed, most neurons in the NPH/MVN (more than 80%) increase their firing rate during eye movements and eye deviations toward the ipsilateral side [32]. Since the majority of them project to the contralateral abducens nucleus [33,34], these burst tonic neurons (IBTNs in Figure 3) must be inhibitory to make their firing functionally comprehensible [32,35]. During straight-ahead fixation, these IBTNs exhibit a substantial tonic firing rate that increases activity during ipsiversive pursuit, possibly driven by excitatory input from neurons in the nucleus of the optic tract (NOT). Their lack of modulation by the motion of the visual field [32] suggests that NOT projections do not originate from background-sensitive neurons [36].

In summary, the horizontal direction of the eyes during fixation is determined by the sustained firing rate of motor and internuclear neurons in the abducens nucleus, which in turn, is determined by a combination of excitatory and inhibitory tonic inputs. Excitatory inputs originate from contralateral EVNs-2 (synapses a or g) and inhibitory inputs from ipsilateral IVNs-1 (synapses i or d) and contralateral IBTNs (synapses k or k’). Any change in the balance of activity between these tonic inputs (and possibly others) is expected to slowly move the eyes horizontally. 

We shall examine next the inputs of anterior and posterior canals to the extraocular motor neurons.

### 3.2. Inputs from Anterior Semicircular Canals

**Figure 4:**
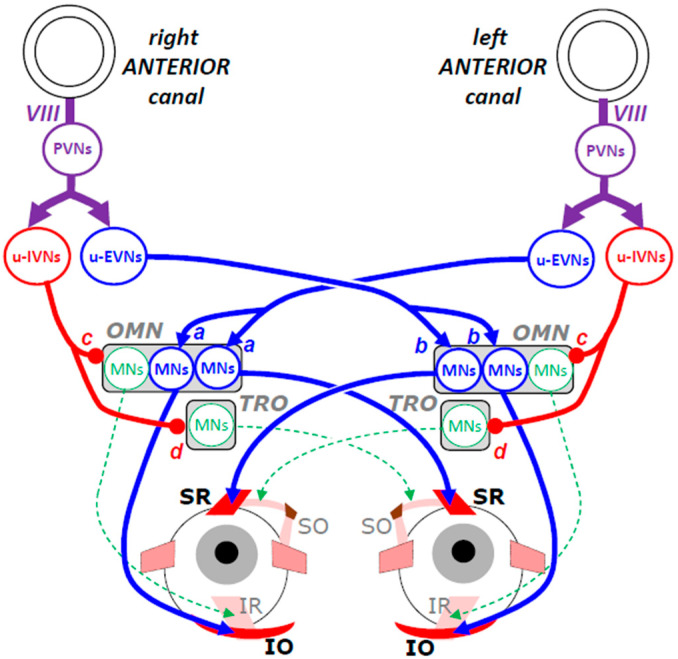
Schematic representation of the neuronal network involved in generating an upward eye movement while the head moves downward. Connecting lines ended by an arrow indicate excitatory connections; those ended by a circle indicate inhibitory synaptic connections. Blue color indicates the agonist neuronal oculomotor elements and red color the antagonist ones. From top to bottom, PVNs: primary vestibular neurons, IVNs: inhibitory vestibular neurons, EVNs: excitatory vestibular neurons, OMN: oculomotor nucleus, TRO: trochlear nucleus, MNs: motor neurons, SR: superior rectus, SO: superior oblique, IR: inferior rectus, IO: inferior oblique. Explanations in the main text. Figure modified and upgraded from [3].

The neuronal connectivities involved in the generation of downward and upward slow eye movements [37] are not as symmetrical as the connectivity subtending the generation of leftward and rightward movements. Figure 4 illustrates the neural network that maintains gaze direction during head pitching and upward eye rotation.

As explained earlier (Figure 2B), the upward eye movement is the outcome of contracting SR and IO muscle fibers. Driven by the increased firing of PVNs connected with the stereocilia in the left anterior canal, excitatory vestibular neurons (u-EVNs) in the left MVN activate the motor neurons innervating the left SR and the right IO muscle fibers (synapses a). Note that this unilateral vestibular stimulation leads to bilateral commands and moves both eyes. However, in addition to rotating both eyes upward, the contraction of these fibers causes an incycloduction of the left eye and an excycloduction of the right eye, as if both eyes were counteracting a tilt of the head toward the left shoulder. This clockwise torsion of both eyes is counterbalanced by the torsion caused by the excitatory input from u-EVNs in the right SVN. Indeed, pitching the head down also stimulates PVNs in the right side and thus increases the firing rate of ipsilateral u-EVNs, which in turn, excites the motor neurons that innervate the right SR and the left IO muscle fibers (synapses b), causing the right eye’s incycloduction and left eye’s excycloduction, respectively. However, the absence of torsion during strictly upward eye movements implies that the outputs of the left and right vestibulo-oculomotor channels have been adjusted so that the torsional signals balance out.

Regarding the antagonist muscles (SO and IR), their relaxation is promoted by u-IVNs, which in the superior vestibular nuclei, inhibit the motor neurons innervating the IR of the ipsilateral eye (synapses c) and those innervating the SO muscle of the contralateral eye (synapse d). 

Contrary to the network involved in the horizontal vestibulo-ocular response (Figure 3), the neural mechanisms triggering the counter-rotation of the eyes when the head pitches down are not completely elucidated. Further investigations are needed. For the sake of our synthesis, we shall keep in mind that the inputs are necessarily bilateral and balanced if the eyes move by the same amount and their torsional consequences cancel.

### 3.3. Inputs from Posterior Semicircular Canals

**Figure 5:**
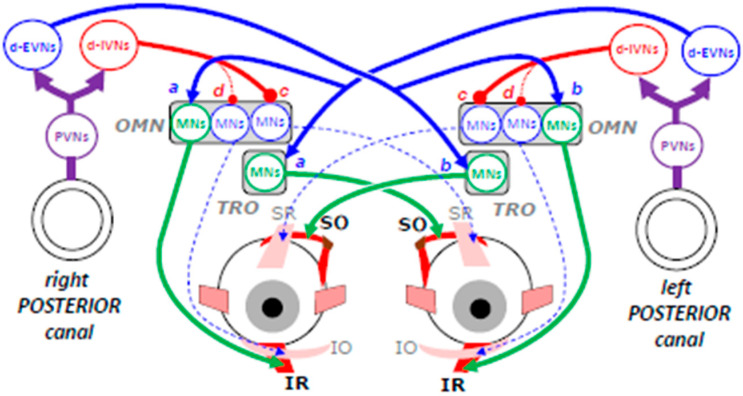
Schematic representation of the neuronal network involved in generating a downward eye movement while the head moves upward. Connecting lines ended by an arrow indicate excitatory connections; those ended by a circle indicate inhibitory synaptic connections. Blue color indicates the agonist neuronal oculomotor elements and red color the antagonist ones. From top to bottom, IVNs: inhibitory vestibular neurons, EVNs: excitatory vestibular neurons, OMN: oculomotor nucleus, TRO: trochlear nucleus, MNs: motor neurons, PVN: primary vestibular neurons, SR: superior rectus, SO: superior oblique, IR: inferior rectus, IO: inferior oblique. Explanations in the main text. Figure modified and upgraded from [3].

Figure 5 schematizes the neuronal network involved in moving both eyes downward while the head tilts upward. The contraction of IR and SO muscle fibers is driven by the increased firing of PVNs connected with the left posterior canal. Excitatory vestibular neurons (d-EVNs) in the left medial vestibular and ventral lateral vestibular nuclei activate the motor neurons innervating the right IR and left SO muscle fibers (synapses a).

As explained earlier (Figure 1B), in addition to depressing both eyes, the contraction of these fibers causes an excycloduction of the right eye and an incycloduction of the left eye, as if the eyes were counteracting a tilt of the head toward the left shoulder. Once again, this binocular torsion is counterbalanced by torsions in the opposite direction and caused by the excitatory input from the right posterior canal to the motor neurons innervating the left IR and the right SO muscle fibers (synapses b). Inhibitory vestibular neurons (d-IVN) located in the superior vestibular nuclei prevent the antagonist muscles from contracting by inhibiting the motor neurons innervating the SR of the contralateral eye (synapses c) and possibly also the motor neurons innervating the IO muscle of the ipsilateral eye (synapse d). As for the neural mechanisms generating upward slow eye movements, the neural mechanisms triggering the counter-rotation of the eyes when the head pitches up are not completely identified. Nevertheless, since the eyes do not exhibit any torsional component, the premotor commands must once again be considered as bilateral and balanced.

### 3.4. Gaze Direction as Equilibrium of Vestibulo-Oculomotor Commands

**Figure 6:**
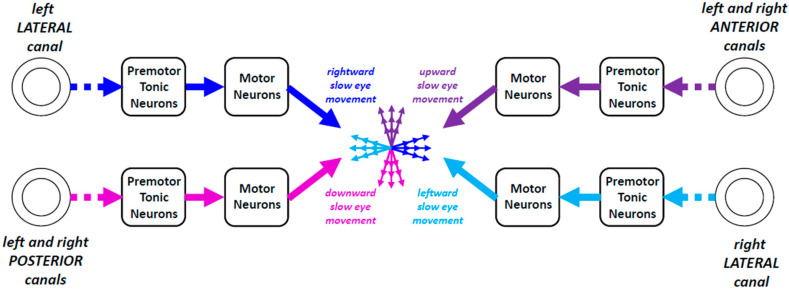
Eye orientation as equilibrium of vestibulo-oculomotor commands. The action potentials conveyed by the neuronal channels linking the left lateral canal to the motor neurons in the right abducens nucleus do not elicit a rightward slow eye movement if they are counterbalanced by the action potentials conveyed by the channels linking the right lateral canal to the motor neurons in the left abducens nucleus and vice versa. Likewise, the action potentials conveyed by the channels linking the anterior canals to the motor neurons in the oculomotor nucleus do not elicit an upward eye movement if they are counterbalanced by the action potentials conveyed by the channels linking the posterior canals to the motor neurons in the oculomotor and trochlear nuclei and vice versa.

The preceding sections permitted us to realize that the bilateral activity of neurons in vestibular nuclei maintains a kind of equilibrium of commands that cancel each other out until the time when an asymmetry happens and generates a slow eye movement (Figure 6). No movement is made as long as the influences exerted by the action potentials conveyed by all vestibulo-oculomotor channels counterbalance each other. Any imbalance yields a slow eye movement. We shall see next that the premotor neurons underlying the generation of slow eye movements also receive signals from brain regions sensitive to visual motion.

In conclusion, the networks underlying the generation of slow eye movements compensating for a head rotation exhibit complexities that contrast with the simpler solutions designed by human engineers. The neurobiological constraints leading to cancel the torsional components caused by the contraction of supraducting or infraducting muscles remain puzzling. However, our description of the connectivity is far from being complete. Indeed, eye movements can be produced in response to “unusual” combinations of head rotations and visual stimulation. Experiments performed with cats showed that the visual stimulation exerts a powerful influence on the generation of vestibulo-ocular responses. After training sessions combining horizontal motions of the visual field with each vertical head movement, horizontal compensatory eye movements can be elicited in the dark whenever the head moves vertically [38].

Finally, contemporary knowledge leads us to consider that the channels transmitting the vestibular signals to the central brain regions and to the motoneurons innervating the extraocular muscles are less numerous than those involved in transmitting the signals engendered by ganglion cells in the retina of both eyes. However, this epistemological situation may result from reasons related to the ease with which we can experimentally test the vestibular contribution to various kinds of motor and cognitive skills. In addition to classical functions related to visuo-postural stabilization, spatial orientation, and navigation [16,17,18], future research might unveil unsuspected cognitive outcomes of the brain processing of vestibular signals. We shall now study the influence of visual signals on eye movements.

## 4. Visual Inputs to Extraocular Motoneurons

Slow eye movements akin to those observed during the vestibulo-ocular reflex are generated when electrical microstimulation is applied to brain regions containing neurons sensitive to the motion of the visual field or to the motion of an object in the external world, i.e., to the sequential excitation of neighboring photoreceptors in the retina. The electrically evoked slow eye movement is interrupted by a quick eye movement in the opposite direction. This rapid movement seemingly prevents the eyes from reaching extreme deviations in their orbit and getting stuck there. Also present during the continuous rotation of the head or during the continuous motion of the visual field, this sequence of slow eye movements followed by quick saccades in the opposite direction is called a nystagmus. The vestibulo-ocular nystagmus is driven by asymmetric vestibular signals, whereas the optokinetic nystagmus is driven by asymmetric visual motion signals. If the slow eye movement is caused by recruitment of inhibitory neurons (as during microstimulation of Purkinje cells in the paraflocculus), the quick eye movements may result from its cessation and a kind of post-inhibitory rebound depolarization of excitatory neurons. 

The neuronal circuits involved in generating these two types of nystagmus do not seem to be affected by the bilateral removal of frontal eye fields and superior colliculi [39], two major brain regions involved in quickly orienting the gaze toward the location of a visual event. The independence of these two regions with respect to the generation of a nystagmus is consistent with the fact that their sustained electrical stimulation does not evoke a nystagmus but a sequence of saccades in the same direction or a long, slow eye movement. By contrast, a nystagmus can be artificially elicited when a long train of electrical pulses is applied in the nucleus of the optic tract [40,41], the NPH [30,31], the vestibular nuclei [30], the flocculus [42,43], the nodulus, and the uvula [44]. The direction of slow phases is ipsilateral when the stimulation is applied in the NPH [31] or NOT [40,41] and contralesional when it is applied more laterally in the vestibular nuclei [30]. 

### 4.1. Nuclei of the Optic Tract and Visual Fixation

The nucleus of the optic tract (NOT) receives a substantial number of afferents from both retinas with a slight contralateral predominance [45,46]. It also receives monosynaptic input from neurons of motion-sensitive areas located in the posterior part of the superior temporal sulcus (STS; [47]). The NOT participates in the generation of slow eye movements by the projections of its neurons to the NPH [48]. It contains cells whose resting activity is relatively high [36,41,49]. Their firing rate increases during ipsilateral pursuit eye movements and decreases during contralateral ones. Consequently, neurons in the left and right NOT emit action potentials when the gaze remains still. A slow ipsilateral eye movement is elicited when neurons on one side emit more action potentials than neurons on the opposite side. Thus, unilateral lesion of NOT causes a nystagmus with contralesional slow eye movements [50,51,52,53], i.e., a nystagmus comparable to the one evoked by the excitation of neurons in the opposite NOT [40,41].

A nystagmus with contralesional slow phases is also engendered after injections of ibotenic acid in the STS [54], which contains neurons projecting to the ipsilateral NOT [47]. After unilateral lesions of area 7 of the parietal cortex [55], a nystagmus appears when the monkeys are placed in the dark. The absence of nystagmus in a lightened environment indicates that visual signals cancel the contralesional nystagmus. Such a compensation does not happen when a small amount of muscimol is injected into one NOT [52,53]. Thus, during lesions in the STS, the asymmetrical cortical input to NOT neurons leads to more neuronal activity in the contralesional NOT than in the ipsilesional NOT, leading to a nystagmus with slow phases directed to the contralesional side. Because of the absence of visual input, neurons in the opposite NOT cannot counteract the contralesional drift of the eyes.

**Figure 7:**
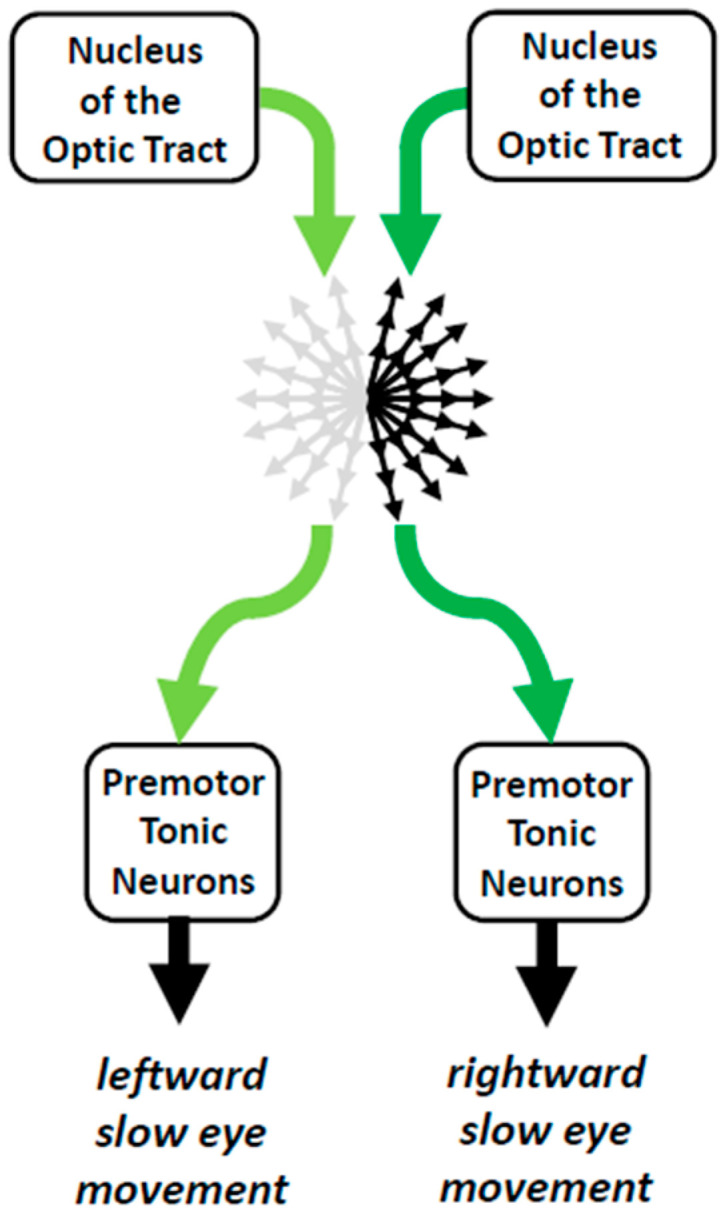
Poly-equilibrium hypothesis. A slow eye movement is not initiated as long as the visuo-oculomotor system is at equilibrium, i.e., as long as opposing commands (issued, for instance, by the left and right nuclei of the optic tract) counterbalance each other. For generating pursuit eye movements, symmetry breaking occurs at the level of premotor neurons located in the nucleus prepositus hypoglossi and vestibular nuclei.

In summary, bilateral activity of neurons in each NOT seems to maintain a kind of equilibrium of commands that cancel each other out until the time when an asymmetry generates a slow eye movement (Figure 7). This bilateral activity does not need equal population activity between the left and right NOTs. Indeed, the efficacy of action potentials from NOT neurons for triggering an ipsilateral movement of the eyes depends not only upon the number of synaptic contacts with their post-synaptic neurons in NPH but also upon the prior activity of targeted neurons and their sensitivity to afferent signals.

Another source of visual signals to motoneurons innervating the extraocular muscles comes from neurons in the intermediate and deep layers of the superior colliculus (dSC), two to three synapses upstream. Their burst of action potentials is tightly linked with the onset of a saccadic eye movement [56] or a saccadic gaze shift [57]. Before studying how dSC activity was found to also participate in visual fixation, we shall report a series of preliminary observations, which led to the conjecture that the bilateral dSC might generate an equilibrium of commands of the same kind as NOT bilateral activity.

### 4.2. Caudal Fastigial Nuclei and Visual Fixation 

A series of observations led to a radical change in the way visual fixation was conceived. Cases were indeed reported in which animals did not accurately direct their gaze toward the location of a target during fixation. During unilateral inactivation of the caudal fastigial nucleus (cFN), cats direct their gaze and mouth toward a location that is offset with respect to the physical target [58]. A comparable eccentric fixation happens in monkeys when they look at a target located in their central visual field. During the unilateral pharmacological perturbation of cFN neurons, there is a mismatch between the location of the target and the location to which the gaze is directed. This mismatch, called “fixation offset”, is ipsilesional when the firing rate of cFN neurons is inhibited by the local injection of muscimol (Gaba-A agonist) [59,60,61,62] and contralesional when it is disinhibited by the local injection of bicuculline (Gaba-A antagonist) [63]. The offset induced by the muscimol injection is a gaze-related disorder and not a defect in positioning the eyes in the orbit, because its magnitude is similar during fixation with the head restrained and when the head is free to move [64]. Moreover, when the head is unrestrained, a large ipsilesional deviation also affects its orientation, implying that the eyes in their orbit are deviated toward the contralesional side [64]. If the offset was a defect in positioning the eyes in the orbit, their deviation should not become contralesional when the head is free to move. Finally, a weakening of the fixation strength accompanies the offset insofar as the monkeys have difficulties in maintaining their gaze directed near the central target when other behaviorally relevant targets are presented in the peripheral visual field. During delayed oculomotor tasks, the animals make frequent irrepressible saccades toward their location (our unpublished observations; see the increased rate of no-go errors in [65]). 

The anatomical fact that, in macaque monkeys, cFN neurons project to the rostral part of both dSC and not to the NPH-MVN [66] is consistent with a gaze-related disorder during fixation rather than an oculomotor impairment. The fastigio-collicular projections are bilateral with a small contralateral predominance. Considering a post-synaptic excitatory influence, the unilateral reduction in cFN input by a local injection of muscimol should lead to an asymmetric distribution of collicular activity with a predominance to the ipsilateral rostral colliculus. If the fixation offset observed during unilateral cFN inactivation results involves the fastigio-collicular projections, then the injection of a small amount of muscimol in the rostral dSC should also lead to an ipsilesional fixation offset. This prediction was confirmed during the experiments in which the involvement of rostral SC in the generation of microsaccades was tested: after injecting a small amount of muscimol, the monkeys exhibited an ipsilesional offset while they fixated a central static target [67] or while they tracked a moving one [68].

### 4.3. Superior Colliculi and Visual Fixation

Despite numerous observations [67,68,69,70] that led to seriously questioning this conjecture, some authors persist in considering the dSC as composed of two antagonistic zones [71,72,73,74]: a "fixation zone" located in its rostral part and a "saccade zone" located more caudally [75,76]. According to them, the “fixation zone” would contain neurons involved in maintaining the direction of the gaze toward a foveal target, whereas neurons in the “saccade zone” would emit a burst of action potentials during saccades toward targets located in the peripheral visual field. In the rostral part of dSC, the activity of neurons would sustain visual fixation by preventing the generation of saccades in two ways: (i) by inhibiting the activity of neurons in the “saccade zone” and (ii) by exciting a group of cells located in the nucleus raphe interpositus (RIP), called omnipause neurons, whose sustained firing rate is interrupted whenever a saccade is made. Thus, during the intersaccadic intervals, the omnipause neurons would inhibit the saccade-related premotor burst neurons in the pontine and mesencephalic reticular formation, whereas their transient pause would disinhibit them, permitting them to emit a burst of action potentials and move the eyes in a saccadic manner. 

Three sets of observations supported the antagonism between putative “fixation” and “saccade” systems. First, neurons in the rostral dSC exhibit a firing rate that is sustained when a visual target is being fixated and that declines during saccades [77,78,79]. Second, low-frequency electrical microstimulation applied in the rostral dSC delays the triggering of saccades toward peripheral targets [76]. Finally, during delayed saccade tasks, monkeys make irrepressible saccades to the location of the peripheral target despite prior training to wait for the central target to be extinguished before looking at the other target [76]. 

Subsequently, several discoveries challenged this antagonism between a set of rostral neurons involved in fixation versus a set of saccade-related neurons, more caudally in the dSC. First, the pre-saccadic firing rate of “fixation” neurons did not differ from the prelude activity of some saccade-related neurons located more caudally [80]. Second, in the rostral superior colliculus, neurons were found that emitted a burst of action potentials during miniature saccades, also called microsaccades [69,70] or fixational saccades [81]. Third, in addition to reducing the frequency of fixational saccades, injection of an inhibitory pharmacological agent (muscimol) in the rostral dSC does not decrease but increases the latency of saccades to peripheral targets [67]. Fourth, during visual fixation, the sustained firing rate does not involve only neurons in the rostral dSC but also neurons located more caudally [82]. Finally, studies that tested the consequences of lesioning or inactivating the RIP did not observe changes in the latency of saccades [83,84].

A saccade is caused by the burst of action potentials emitted by motoneurons innervating the agonist extraocular muscles. This burst is the outcome of presynaptic input from excitatory and inhibitory premotor neurons. Two groups of premotor neurons have been identified: short-lead (or medium-lead) and long-lead burst neurons [85,86]. The neurons of each group emit a burst of action potentials during saccades, but long-lead burst neurons (LLBNs) differ from the other neurons (SLBNs or MLBNs) by a prelude activity preceding the saccade-related burst. Excitatory LLBNs and SLBNs are intermingled in the ipsilateral pontine reticular formation, whereas inhibitory LLBNs and SLBNs are intermingled in the contralateral medullary reticular formation. In turn, the firing rate of these premotor neurons is driven by spikes emitted by neurons in the deep layers of the superior colliculus (dSC) and the caudal fastigial nucleus (cFN). The two categories of short-lead and long-lead burst neurons have also been found in the dSC and called burst neurons and build-up neurons, respectively [87]. In the dSC, saccade-related neurons emit a burst shortly before small saccades, whereas those located more caudally emit a burst of action potentials shortly before larger saccades [70,80]. When fixating a target located in the central visual field, neurons in the left and right rostral SC exhibit a sustained firing rate, as do neurons located more caudally [80]. The emitted action potentials are transmitted to the premotor burst neurons without eliciting a saccade, either because they excite omnipause neurons (OPNs), which inhibit burst neurons, or because they recruit as many EBNs as IBNs. The first option remains to be verified [88]. According to the second option, a saccade is launched as soon as the EBNs recruit enough IBNs to inhibit the EBNs and IBNs located on the opposite side and whose activity suppresses the motoneurons innervating the antagonist muscles. Thus, prior to the saccade onset, the bilateral activity emitted by neurons in the left and right dSC and cFN maintains a kind of balance of mutually opposing commands. When the central target is deactivated, collicular activity decreases approximately 100 ms after [78,89], eliminating the procrastination engendered by the antagonistic opposition and giving way to saccades with shorter latency than when the central target remains present. According to this scenario, suppressing the activity of neurons in the rostral dSC by the local injection of muscimol should decrease the latency of ipsilesional saccades, a prediction that has been confirmed [67,76]. The latency of contralesional saccades may not be reduced. It can indeed be increased if muscimol suppresses the activity of a part of the neurons of the population involved in their generation [90] and makes them slightly hypometric [67].

**Figure 8:**
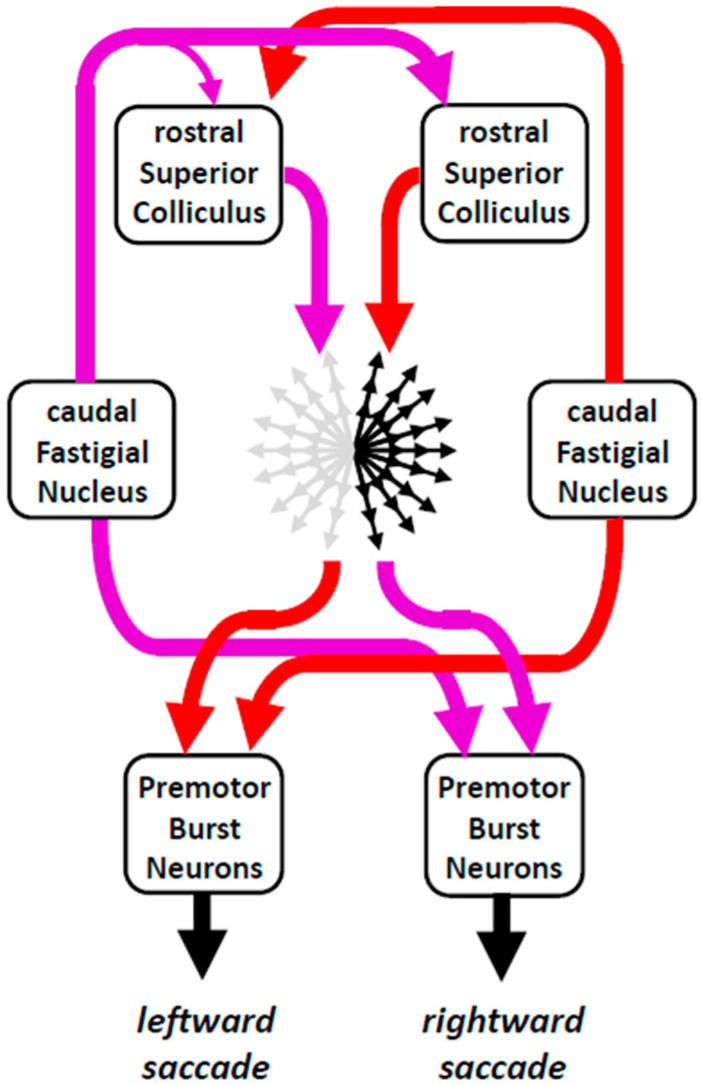
Poly-equilibrium hypothesis. A saccade is not initiated as long as the visuo-oculomotor system is at equilibrium, i.e., as long as opposing commands (issued, for instance, by neurons in the left and right rostral superior colliculi) counterbalance each other. For generating saccadic eye movements, symmetry breaking involves premotor neurons located in the mesencephalic reticular formation for vertical saccades and in the paramedian pontine reticular formation for horizontal saccades.

In summary, rather than being composed of two distinct zones, the deep superior colliculus forms a continuum in which the population of active neurons depends on the location of the target in the visual field, with rostral regions containing cells sensitive to targets located in the central visual field and caudal regions containing cells sensitive to targets located in the peripheral field. The action potentials emitted by dSC neurons are transmitted to two neural networks involved in the generation of horizontal and vertical saccades, respectively. Fixation then corresponds to a kind of equilibrium state in which the activity distributed over the left and right colliculi determines the direction that the gaze takes, in the absence of commands to make saccades (Figure 8).

Microsaccades result from temporary imbalances between the fluctuations of signals emitted by neurons in the two deep superior colliculi. Concerning the collicular projections to omnipause neurons, it remains unclear whether they are specifically involved in visual fixation, because their inactivation causes neither instability of gaze during target fixation nor premature triggering of saccades.

### 4.4. Additional Evidence from Clinical and Behavioral Studies

Prior to these inactivation experiments in the cFN and rostral dSC of monkeys, fixation offsets had been reported after unilateral ablation of their frontal eye field (FEF) [91,92] or when a small amount of muscimol was injected within it [93]. By contrast, lesions in parietal areas of the cerebral cortex, which project both to the ipsilateral deep superior colliculus [94,95] and FEF [96,97,98], do not lead to offsets during visual fixation [99,100,101]. 

However, human patients suffering from acute cerebral vascular accidents in one cerebral hemisphere exhibit a conjugate ipsilesional deviation of their eyes [102,103] and head [104], even when they explore a visual scene. An asymmetric distribution in the amplitude of fixational saccades toward the blind visual field has also been reported in patients suffering from homonymous hemianopia; some of them exhibit eccentric fixation [105].

Offsets in gaze direction during fixation of a visual target are not restricted to lesional or pathological cases. In a completely dark visual environment (scotopic conditions), rhesus macaques exhibit an upward offset when they look at a small visual target [106,107,108]. Their gaze is directed above the location fixated in photopic conditions. Asymmetrical representations of the upper and lower visual fields [109,110] may account for this offset. They may also explain the upward deflection of saccades toward targets located along the horizontal meridian [108]. In darkness, this asymmetry does not affect the population of active neurons. However, in photopic conditions, more cells sensitive to the lower visual field may be recruited and counteract the upward bias caused by the asymmetrical representation of the upper and lower visual fields. Thus, in scotopic conditions, the absence of their recruitment causes the upward fixation offset and the upward deflection of horizontal saccades. Asymmetrical neuronal representations of the upper and lower visual fields can also explain the downbeat nystagmus exhibited by some patients if a neuronal death targets the cells involved in compensating for the asymmetry [111,112].

## 5. Conclusions: The Poly-Equilibrium Theory

Based upon the synthesis presented in the preceding paragraphs, the idea that fixation is a state during which the generation of eye movements is inhibited should be reevaluated. Instead, a seemingly counterintuitive view can be proposed according to which the absence of movement results from the combination of multiple antagonistic commands counterbalancing each other. In fact, this view extends to a bilateral mode the coding of saccade amplitude and direction by the population of active neurons in the dSC [90]. Building on previous syntheses [3,53,113], the concept of gaze direction as equilibrium can now be generalized to other eye movements and be replaced by, or rather introduce, the novel concept of “poly-equilibrium” [3,88].

An imbalance of activity between the left and right SC favors the generation of saccades toward the side opposite the most active SC. In contrast, an imbalance of activity between the left and right NOT favors a slow eye movement toward the side of the most active nucleus. The contrast of laterality is indicated by the direction of eye movements evoked by electrical microstimulation. Saccades evoked by collicular stimulation are directed toward the contralateral side [114,115,116]; slow eye movements evoked by stimulation of the nucleus of the optic tract are directed toward the ipsilateral side [40,41]. The contrast of laterality is also consistent with the results of single-unit recording studies. The firing rate of neurons in NOT increases during ipsilateral pursuit eye movements and decreases during contralateral ones [36,41,47], whereas SC neurons emit a burst of action potentials during contralateral saccades [70,114,117,118].

The notion of poly-equilibrium is more appropriate than that of equilibrium because multiple equilibria operate in parallel and simultaneously during visual fixation. They involve action potentials conveyed by (i) the visuomotor channels responsible for the generation of saccades, (ii) the sensorimotor channels responsible for the generation of slow eye movements from unbalanced visual, vestibular and proprioceptive cervical signals, (iii) those responsible for the approach response (accommodation and vergence), and (iv) those that maintain the head upright [53]. The tonic activities are distributed among different groups of neurons in the left and right parts of the reticular formation, cerebellum, deep superior colliculi, and cerebral cortex. An asymmetry within the set of commands participating in this poly-equilibrium modifies the direction of gaze during the visual fixation if it is not counterbalanced downstream.

Prior to the onset of a saccadic or slow eye movement, the sensory signals do travel toward the motor neurons through a large network of cells that already evince a spontaneous, sustained firing rate, which maintains the static orientations of the eyes and head. These tonic premotor activities form an equilibrium of commands that counterbalance each other. Thus, gaze direction stays relatively stable during visual fixation, occasionally interrupted by saccadic intrusions or by ocular drifts. These interruptions are caused by asymmetric changes in the contraction of extraocular muscles. For generating saccadic and slow eye movements, the imbalance involves different groups of neurons in the left and right parts of the brainstem. Saccades involve the recruitment of premotor burst neurons located in the pontomedullary reticular formation, whereas slow eye movements involve the recruitment of premotor tonic neurons in the vestibular nuclei. 

Considering the direction of gaze as a poly-equilibrium leads to conceiving the imbalance between “commands” and “anti-commands” as a general trigger mechanism for initiating an eye movement. Depending upon the sensorimotor and motivational contexts, these “commands” and “anti-commands” are conveyed by neurons whose number can vary considerably. Then, the oculomotor reaction time corresponds to the duration for breaking their balance [119]. Rooted in the basic neurophysiology of eye movements, the poly-equilibrium theory offers an alternative to cognitivist models of decision making, which rely on metaphors such as “winners take all” or “accumulators”. 

## Data Availability

No new data were created or analyzed in this epistemological review of the literature about the neurophysiology of eye movements.

## Abbreviations

ABD	abducens nucleus
AINs	abducens internuclear neurons
cFN	caudal fastigial nucleus
EVNs-1(2)	excitatory vestibular neurons of type 1 (type 2)
FEF	frontal eye field
IBTNs	inhibitory burst tonic neurons
IO	inferior oblique
IR	inferior rectus
IVNs-1(2)	inhibitory vestibular neurons of type 1 (type 2)
LR	lateral rectus
MNs	motoneurons
MR	medial rectus
MVN	medial vestibular nucleus
NOT	nucleus of the optic tract
NPH	nucleus prepositus hypoglossi
OMN	oculomotor nucleus
PVNs	primary vestibular neurons
PVP	position vestibular pause neurons
RIP	raphe interpositus
SC	superior colliculus
SO	superior oblique
SVN	superior vestibular nucleus
TRO	trochlear nucleus
VOR	vestibulo-ocular response
